# Hereditary angioedema: how to approach it at the emergency department?

**DOI:** 10.31744/einstein_journal/2021RW5498

**Published:** 2021-04-01

**Authors:** Faradiba Sarquis Serpa, Eli Mansour, Marcelo Vivolo Aun, Pedro Giavina-Bianchi, Herberto José Chong, Luisa Karla Arruda, Regis Albuquerque Campos, Antônio Abílio Motta, Eliana Toledo, Anete Sevciovic Grumach, Solange Oliveira Rodrigues Valle

**Affiliations:** 1 Escola Superior de Ciências Santa Casa de Misericórdia de Vitória VitóriaES Brazil Escola Superior de Ciências, Santa Casa de Misericórdia de Vitória, Vitória, ES, Brazil.; 2 Faculdade de Ciências Médicas Universidade Estadual de Campinas CampinasSP Brazil Faculdade de Ciências Médicas, Universidade Estadual de Campinas, Campinas, SP, Brazil.; 3 Faculdade Israelita de Ciências da Saúde Albert Einstein Hospital Israelita Albert Einstein São PauloSP Brazil Faculdade Israelita de Ciências da Saúde Albert Einstein, Hospital Israelita Albert Einstein, São Paulo, SP, Brazil.; 4 Faculdade de Medicina Universidade de São Paulo São PauloSP Brazil Faculdade de Medicina, Universidade de São Paulo, São Paulo, SP, Brazil.; 5 Universidade Federal do Paraná CuritibaPR Brazil Universidade Federal do Paraná, Curitiba, PR, Brazil.; 6 Faculdade de Medicina de Ribeirão Preto Universidade de São Paulo Ribeirão PretoSP Brazil Faculdade de Medicina de Ribeirão Preto, Universidade de São Paulo, Ribeirão Preto, SP, Brazil.; 7 Faculdade de Medicina Universidade Federal da Bahia SalvadorBA Brazil Faculdade de Medicina, Universidade Federal da Bahia, Salvador, BA, Brazil.; 8 Faculdade de Medicina de São José do Rio Preto São José do Rio PretoSP Brazil Faculdade de Medicina de São José do Rio Preto, São José do Rio Preto, SP, Brazil.; 9 Faculdade de Medicina do ABC Santo AndréSP Brazil Faculdade de Medicina do ABC, Santo André, SP, Brazil.; 10 Universidade Federal do Rio de Janeiro Rio de JaneiroRJ Brazil Universidade Federal do Rio de Janeiro, Rio de Janeiro, RJ, Brazil.

**Keywords:** Angioedema, Angioedemas, hereditary, Emergencies, C1-inhibitor, Abdominal pain, Laryngeal edema, Asphyxia, Bradykinin

## Abstract

Angioedema attacks are common causes of emergency care, and due to the potential for severity, it is important that professionals who work in these services know their causes and management. The mechanisms involved in angioedema without urticaria may be histamine- or bradykinin-mediated. The most common causes of histamine-mediated angioedema are foods, medications, insect sting and idiopathic. When the mediator is bradykinin, the triggers are angiotensin-converting enzyme inhibitors and factors related to acquired angioedema with deficiency of C1-inhibitor or hereditary angioedema, which are less common, but very important because of the possibility of fatal outcome. Hereditary angioedema is a rare disease characterized by attacks of edema that affect the subcutaneous tissue and mucous membranes of various organs, manifesting mainly by angioedema and abdominal pain. This type of angioedema does not respond to the usual treatment with epinephrine, antihistamines and corticosteroids. Thus, if not identified and treated appropriately, these patients have an estimated risk of mortality from laryngeal edema of 25% to 40%. Hereditary angioedema treatment has changed dramatically in recent years with the development of new and efficient drugs for attack management: plasma-derived C1 inhibitor, recombinant human C1-inhibitor, bradykinin B2 receptor antagonist (icatibant), and the kallikrein inhibitor (ecallantide). In Brazil, plasma-derived C1 inhibitor and icatibant have already been approved for use. Proper management of these patients in the emergency department avoids unnecessary surgery and, especially, fatal outcomes.

## INTRODUCTION

Angioedema is characterized by non-inflammatory, localized, asymmetric, disfiguring, and self-limited swelling of the deep dermis, subcutaneous, and/or submucosal tissue, resulting from vasodilation and an increase in vascular permeability.^(1)^

Due to potential severity, especially when angioedema affects the airways, the therapeutic approach in emergency department is often initiated soon after admission, before taking the medical history and possible identification of the etiology. However, the recognition and differentiation of the different etiologies and pathogenesis of angioedema are important for an efficacious treatment in the emergency department.^(2,3)^

The mechanism involved in angioedema without urticaria can be mediated by histamine or bradykinin. The most common causes of histaminergic angioedema are food, medication, insect bites, and idiopathic. In bradykinin-mediated angioedema, the most frequent triggers are angiotensin-converting enzyme inhibitors (ACEi) and factors related to hereditary angioedema (HAE) or acquired angioedema (AAE). Although less common, AAE and HAE are relevant because of the possibility of a fatal outcome, if not properly treated.^(4)^

Hereditary angioedema is a rare, potentially fatal, and still underdiagnosed disease. Its attacks can affect both the dermis and subcutaneous, as well as internal organs - predominantly the gastrointestinal tract and upper airways - the most feared outcome being laryngeal angioedema. It is estimated that 25% to 40% of HAE attacks lead to death by asphyxia, when untreated.^(5)^ Intestinal wall edema is frequent and disabling, and abdominal pain may be the only manifestation during an attack. Abdominal angioedema attack may be misdiagnosis of acute abdomen leading to unnecessary surgery.^(6)^

In the emergency department, HAE attacks can be mistaken with other types of angioedema, mainly histaminergic. A Brazilian study showed that 29% of patients with HAE reported deaths in family members due to asphyxia.^(7)^ In the cases studied by Bork et al.,^(5)^the cause of death in one third of patients was asphyxia, those with no confirmed diagnosis being most at risk.

Data from the United States show that approximately 110 thousand emergency department visits per year are due to HAE or AAE. These patients have a higher hospital admission rate than those treated for allergy-caused angioedema.^(8)^In an Italian study, 0.37% of patients admitted to the emergency department during a six month period presented with angioedema of varyng causes.^(9)^ In Canada, 0.1% of emergency care was due to angioedema of different etiologies, and more than one third of patients reported previous visits for the same cause.^(10)^ No data is avaiable in Brazil on the number of visits to emergency department caused by angioedema attacks.

There is an urgent need to inform health professionals, especially those working in emergency department about HAE as well as relevant aspects for the care of angioedema attacks. The authors aim to address these questions.

## METHODS

We evaluated publications that addressed the topic HAE through revision of MEDLINE^®^ (PubMed^®^), Cochrane, and Scientific Electronic Library Online (SciELO) databases, and considered the consensus and guidelines previously published, with the purpose of using practices already approved by experts on the subject, including Brazil, over the last 10 years. The Health Sciences Descriptors (DeCS) “hereditary angioedema”, “emergency” and “bradykinin,” the Boolean operator “AND” and their correspondents in Portuguese were used.

### Classification of angioedema without urticaria

**Figure 1:**
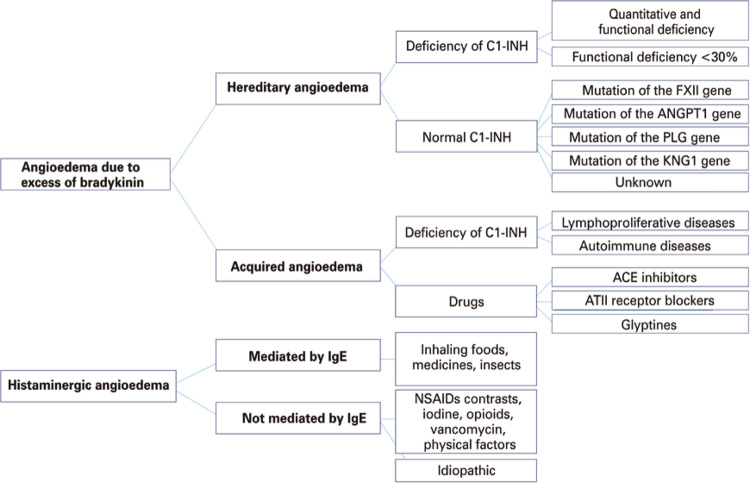
Classification of angioedema in terms of associated mechanisms and defects

C1-INH: C1 inhibitor; FXII: coagulation factor XII; ANGPT1: angiopoietin-1; PLG: plasminogen; KNG1: kininogen 1; ACE: angiotensin-converting enzyme; ATII: angiotensin II; IgE: immunoglobulin E; NSAIDs: non-steroid anti-inflammatory drugs.

Angioedema can be classified in relation to the vasoactive mediator causing the edema, *i.e.*, histamine, or bradykinin. This classification allows the identification of the type of angioedema and its appropriate treatment^(3)^ (Figure 1).

Histaminergic angioedema is the most common. It progresses rapidly,^(4)^is generally associated with urticaria, and occurs due to mast cell degranulation, mediated or not by immunoglobulin E (IgE), or by alteration in arachidonic acid metabolism, typified by cases induced by nonsteroidal anti-inflammatory drugs (NSAIDs).^(11)^

Bradykinin-mediated cases are clinically characterized by the absence of urticaria and include HAE, C1-inhibitor AAE (C1-INH), and drug-induced: ACEi, angiotensin II receptor blocker, and glyptins.

There are two subtypes of HAE: due to deficiency of C1-inhibitor (HAE-C1-INH) and with normal C1-INH (HAE with normal C1-INH).^(1,12)^The genetic inheritance of HAE-C1-INH is autosomal dominant, resulting from mutations in the SERPING1 gene, which encodes a serinoprotease, C1-INH, with several sites of action. Such mutations result in quantitative and/or functional deficiency of C1-INH, which causes the exaggerated production of bradykinin, with a consequent increase in vascular permeability and the onset of angioedema. Hereditary angioedema with normal C1-INH is associated with other mutations, and also lead to bradykinin overproduction.^(13,14)^ Cases of coagulation factor XII (FXII) mutation are the most common.^(13,14)^Other recently described mutations occur in plasminogen (HAE-PLG),^(15)^angiopoietin-1 (HAE-ANGPT1),^(13)^ and kininogen-1 (KNG1) genes.^(16)^

AAE-C1-INH results from C1-INH consumption, and the most common causes being lymphoproliferative and autoimmune diseases.^(17)^The clinical picture is similar to HAE, but the onset of symptoms is later, and the family history is negative.^(1,12)^

Antihypertensive agents in the ACEi group represent 0.1% to 0.5% of angioedema cases seen in emergency department .^(17)^ Attacks can occur regardless of the length of use, and the ACEi most frequently related to the occurrence of angioedema are captopril, enalapril, and lisinopril.^(18)^ Medications of the group of gliptins, sitagliptin, vildagliptin, saxagliptin, alogliptin, and linagliptin, used to treat type 2 *diabetes mellitus,* are also potential triggers of angioedema. These drugs inhibit the enzyme dipeptidyl peptidase 4 (DPP4i), which is important in the degradation of bradykinin and substance P.^(19)^

Data on the prevalence of bradykinin-mediated angioedema are scarce, and studies show that ACEi angioedema is more common than HAE, and the latter being more common than AAE-C1-INH.^(20)^

Some clinical characteristics, such as age of onset of symptoms, positive family history, the association of urticaria, location of the attack, and speed of onset and progression, permit differentiating five main phenotypes of angioedema (Table 1).

**Table 1:** Clinical characteristics of the main phenotypes of angioedema

Clinical characteristics	Main phenotypes of angioedema					
	Histaminergic or allergic angioedema	HAE with C1-INH deficiency	HAE with normal C1-INH	AAE-C1-INH	ACEi	Idiopathic
Age of onset	Variable	Childhood	Adolescent/adult	Adult	Adult	Variable
Family history	Atopy (?)	75% cases	Variable	No	No	No
Sex predilection	No	No	Female	No	No	No
Associated urticaria	Yes/no	No	No	No	No	Yes/no
Attack site	Lips, eyes, tongue, and larynx	Face, tongue, larynx, genitals, limbs, and abdomen	Face, tongue, larynx, genitals, limbs, and abdomen	Face, tongue, larynx, genitals, limbs, and abdomen	Face, tongue, and larynx	Lips, eyes, tongue, and larynx
Onset of attack	Rapid (up to 1 hour)	Slow and gradual (hours)	Slow and gradual (hours)	Slow and gradual (hours)	Slow and gradual (hours)	Variable

Source: adapted from Moellman JJ, Bernstein JA, Lindsell C, Banerji A, Busse PJ, Camargo CA Jr, Collins SP, Craig TJ, Lumry WR, Nowak R, Pines JM, Raja AS, Riedl M, Ward MJ, Zuraw BL, Diercks D, Hiestand B, Campbell RL, Schneider S, Sinert R; American College of Allergy, Asthma & Immunology (ACAAI); Society for Academic Emergency Medicine (SAEM). A consensus parameter for the evaluation and management of angioedema in the emergency department. Acad Emerg Med. 2014;21(4):469-84.^(21)^ HAE: hereditary angioedema; C1-INH: C1 inhibitor; AAE-C1-INH: acquired angioedema with deficiency of C1-inhibitor; ACEi: angiotensin enzyme converter inhibitor.

### Pathophysiology of bradykinin-mediated angioedema

**Figure 2:**
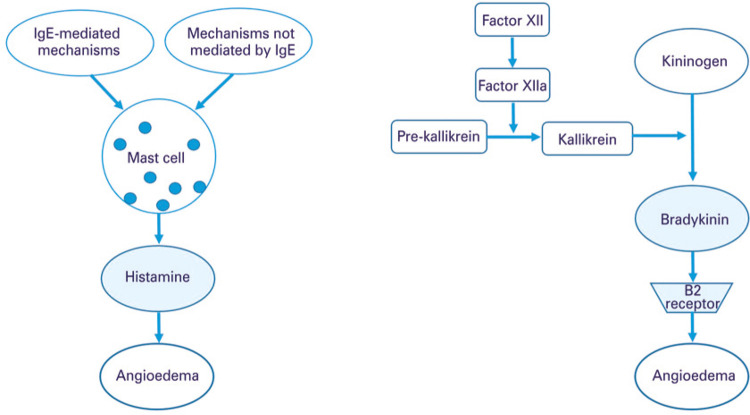
Pathophysiology of (A) histamine-mediated and (B) bradykinin-mediated angioedema, without urticaria

Source: adapted from Bernstein JA, Cremonesi P, Hoffmann TK, Hollingsworth J. Angioedema in the emergency department: a practical guide to differential diagnosis and management. Int J Emerg Med. 2017;10(1):15. Review.^(3)^

Bradykinin is the primary mediator in AAE and HAE with quantitative or functional deficiency of C1-INH. C1-INH regulates several proteins of the complement, contact, coagulation, and fibrinolysis systems. The activation of the contact system begins with the activated FXII that converts pre-kallikrein into kallikrein; this, in turn, cleaves the high molecular weight kininogen (HK), leading to the release of bradykinin. This kinin binds to its B2 receptor, constitutively expressed in endothelial cells, and interferes with endothelial junctions, increasing vascular permeability and causing angioedema. In C1-INH deficiency, the lack of control of the contact system activation is more relevant to the release of bradykinin. On the other hand, by activation of the complement system, the plasma C4 levels decrease due to lack of C1-INH, and the dosage of C4 is used as a screening test for HAE^(1,22)^ (Figure 2).

IgE: immunoglobulin E; Factor XIIa: activated factor XII; B2 receptor B2: bradikinin receptor.

In HAE with normal C1-INH, the mutations in FXII result in greater activation of FXII by plasmin, generating the activated factor XII, which acts on pre-kallikrein.^(13,14)^ Thus, FXII mutant activates rapidly after cleavage by plasmin and escapes from C1-INH inhibition. In cases of HAE with mutations in PLG,^(15)^ ANGPT1,^(13)^ or KNG1 genes,^(16)^ other mechanisms are involved. The PLG, *eg.,* is a circulating zymogen, which is converted into the active plasmin enzyme by cleavage of a peptide bond. Plasmin, the main enzyme of fibrinolysis seems to be involved in the pathogenesis of HAE with normal C1-INH, but the role of the fibrinolytic system in HAE has yet to be fully clarified.^(15)^ In case of mutations in the ANGPT1 gene, the loss of protein function is described, with consequent reduction in the angiopoietin-1 receptor activation capacity, expressed in endothelial cells. This, in turn, would influence vascular permeability.^(13)^ In KNG1 gene mutation, an aberrant bradykinin formation occurs, with a longer half-life and, consequently, more active.^(16)^

### Clinical and laboratory diagnosis

When treating patients with angioedema, a detailed medical history, physical examination, and response to conventional treatment with antihistamines (anti-H1), corticosteroids (EC), and epinephrine may indicate the possible etiology.

The presence of urticaria associated with angioedema, both in the current episode and in previous attacks, strongly suggests histaminergic angioedema.^(17)^ This type of angioedema evolves rapidly,^(6)^ and the most common triggers being medication, food, insect venom, physical (heat, cold, and pressure), and even idiopathic factors. In these situations, the removal of the trigger and conventional treatment are effective.^(17)^

When other organs or systems are involved, configuring anaphylaxis, the serum tryptase dosage may corroborate this hypothesis. This test is generally not available in the Brazilian emergency departments, and the delay for the result does not help assistance in the emergency setting. High level of tryptase during the attack will confirm degranulation of mast cells. On the other hand, the normal dosage of tryptase during the attack does not exclude anaphylaxis.^(21)^ High tryptase does not occur in bradykinin-induced angioedema, such as HAE, AAE-C1-INH, or ACEi-induced.

Although angioedema attacks induced by ACEi, AAE-C1-INH, and HAE, all mediated by bradykinin, usually take hours to become full blown, there are reports of sudden events.^(21)^ In addition, they do not present associated urticaria and do not respond to conventional treatment.^(1,17)^

The diagnosis of HAE is generally not made in emergency department. Diagnosis requires clinical data such as family history of angioedema, edema persisting for 3 to 5 days, no response to conventional treatment, recurrent airway edema, and abdominal pain episodes with no apparent cause, sometimes with a history of unnecessary abdominal surgeries.^(21)^ In some cases, prodrome signs and symptoms may occur, such as *erythema marginatum*, characterized by serpinginous erythematous and non-pruritic lesions, which may be confuses with urticaria.^(1)^ A family history of HAE is an important warning sign, but is absent in approximately 25% of cases.^(17)^

Another relevant aspect of women with HAE is the higher frequency and/or severity of attacks in periods of a higher estrogen level, either endogenous (menstrual cycle, pregnancy), or under exogenous administration (contraceptives or hormone replacement) among women.^(14)^ Hereditary angioedema with normal C1-INH levels is more associated with estrogen, being even more frequent in women.^(14)^

In patients with no other cause of angioedema, but who present any of these indicators, a diagnosis of HAE should be presumed, especially if there is no clinical response to conventional treatment with epinephrin, anti-H1, and CE.^(17)^

The diagnosis of HAE C1-INH is confirmed by quantitative and/or functional evaluation of C1-INH. Laboratory tests are often not available for diagnosis in the emergency department. Nevertheless, the dosage of C4 during the attack may contribute to the diagnosis. It is estimated that almost 100% of these patients have reduced C4 levels during the attack, so, if the level of C4 is normal HAE C1-INH can be ruled out.^(21)^

AAE-C1-INH can be differentiated from HAE-C1-INH as the former, in most cases has low levels of C1q concentration, in addition to the reduction of C4, and quantitative and functional C1-INH.

### Warning signs for hereditary angioedema

Grumach et al.,^(23)^ elected the main warning signs for HAE, based on pathophysiology, signs, and symptoms, laboratory tests, and response to specific therapies. To facilitate memorization, the signs were listed in alphabetical order and are called the ABC of angioedema.^(23)^

With the same objective, the Brazilian Group for the Study of Hereditary Angioedema (Gebraeh) created a mnemonic rule with the warning signs for diagnosis of HAE: HAAAAE.^(1)^ All these signs are listed in table 2.^(1)^

**Table 2:** Warning signs of hereditary angioedema

HAAAAE^(1)^	ABC of angioedema^(23)^
Hereditary	Angioedema
Angioedema [recurrent]	Bradykinin
Abdominal pain	C1 inhibitor
Absence of urticaria	Triggers
Antihistamines with no effect	Epinephrine with no response
Estrogen association	Affected family members
	Gastrointestinal and/or edema of the glottis

Source: Giavina-Bianchi P, Arruda LK, Aun MV, Campos RA, Chong-Neto HJ, Constantino-Silva RN, et al. Diretrizes brasileiras para o diagnóstico e tratamento do angioedema hereditário – 2017. Arq Asma Alerg Imunol. 2017;1(1):23-48;^(1)^ Grumach AS, Ferraroni N, Olivares MM, López-Serrano MC, Bygum A. An ABC of the warning signs of hereditary angioedema. Int Arch Allergy Immunol. 2017;174(1):1-6. Review.^(23)^

### Triggers for hereditary angioedema attacks

Hereditary angioedema attacks can be spontaneous or triggered by factors identified by means of a detailed medical history. The following have been described as triggers: trauma, stress, infection, surgical and dental procedures, menses, gestation, alcohol consumption, extreme change in temperature, physical exercise, use of ACEi, exposure to estrogen, among others.^(1)^ Identification of the triggering factor may contribute towards the diagnosis and future follow-up.

### Treatment of a hereditary angioedema attack

**Figure 3:**
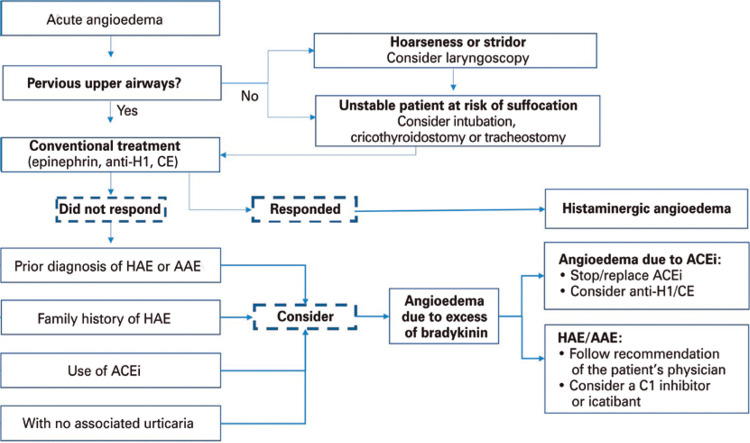
Algorithm for addressing acute angioedema in the emergency department

Anti-H1: antihistaminec; CE: corticosteroid; HAE: hereditary angioedema; AAE: acquired angioedema; ACEi: angiotensin-converting enzyme inhibitor.

The first step when approaching a patient with a HAE attack affecting the airways, tongue, and/or uvula is to keep the airway patent. In unstable patients, with imminent risk of asphyxia, orotracheal intubation (OTI) should not be delayed^(21)^ (Figure 3). It is important to emphasize that in the initial phase of airway obstruction, no drop in oxygen saturation is observed. Emergency room monitoring is indicated and, if cases of hypotension or dehydration, fluid replacement must be initiated.^(24)^

**Figure 4:**
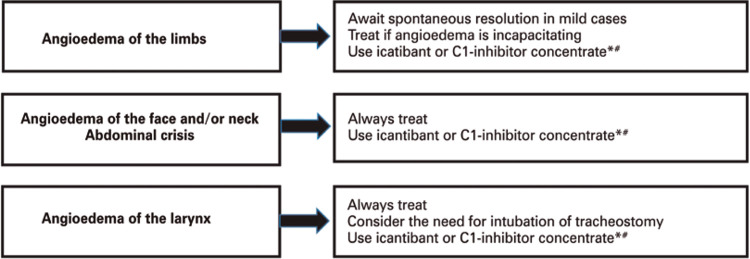
Recommendations for treatment of hereditary angioedema attack, according to the affected area

Treatment of an attack in a patient diagnosed with HAE may vary according to the severity and location of the attack, and requires the use of drugs that act by preventing the action of bradykinin in endothelial cells, or increasing levels of C1-INH, and, consequently, reducing bradykinin levels^(1)^ (Figure 4).

* Available in Brazil: C1-inhibitor concentrate (Berinert^®^) and icatibant (Firazyr^®^); ^#^ in all situations, if C1-inhibitor concentrate or icatibant is not available, use fresh frozen plasma.

Pharmacological treatment has drastically changed in recent years, with the introduction of new and efficient drugs for management of attacks. There are four groups of drugs available: plasma-derived C1-inhibitor concentrate (pd C1-INH), recombinant human C1-inhibitor (Rh C1-INH), bradykinin B2 receptor antagonist (icatibant), and kallikrein inhibitor (ecalantide).^(24-26)^ In Brazil, so far, there are two products approved by the National Health Surveillance Agency (ANVISA - *Agência Nacional de Vigilância Sanitária*) pdC1-INH (Berinert^®^) and icatibant (Firazyr^®^)^(1,27,28)^ (Table 3).

**Table 3:** Characteristics and guidelines for the drugs available in Brazil for attacks

Characteristics/guidelines	Drug	
	Icatibant (Firazyr^®^)	C1-INH concentrate (Berinert^®^)
Age range	>2 years	No age limit
Formulation	10mg/mL of icatibant (syringe with 3mL of the solution)	500IU of C1-INH in freeze-dried powder
Dose	0.4mg/kg up to 18 years 30mg over 18 years	20IU/kg
Administration route	Subcutaneous, slowly Preferentially in the abdominal region	Slow intravenous or infusion (4mL/minute)
Aspect of the solution	Colorless and clear	Colorless and limpid
Storage temperature	2°C to 8°C	2°C to 8°C
Storage after use of fractionated dose or reconstitution	Not recommended Under 18 years and weight of 65kg; the unused volume should be discarded	After reconstitution, exclusively in the ampoule flask
Time of storage	Not recommended	Maximum of 8 hours at room temperature

Source: Brasil. Ministério da Saúde. Agência Nacional de Vigilância Sanitária (ANVISA). Bulário eletrônico. Firazyr: solução injetável. Bula profissional do medicamento. Brasília (DF): ANVISA; 2019 [citado 2020 Jan 22]. Disponível em: https://consultas.anvisa.gov.br/#/bulario/q/?nomeProduto=FIRAZYR;^(27)^ Brasil. Ministério da Saúde. Agência Nacional de Vigilância Sanitária (ANVISA). Bulário eletrônico. Berinert: pó liofilizado para solução injetável. Bula profissional do medicamento. Brasília (DF): ANVISA;2019 [citado 2020 Jan 22]. Disponível em: https://consultas.anvisa.gov.br/#/bulario/q/?nomeProduto=berinert^(28)^ C1-INH: C1-inhibitor.

C1-INH concentrates for intravenous use have been shown to be efficacious and safe in the treatment, both of children and adults, of all forms of HAE attacks due to C1-INH deficiency.^(25,29,30)^ Berinert^®^, approved in Brazil, is indicated for intravenous administration at a dose of 20IU/kg, regardless of the severity of the attack.^(29)^ Another concentrate of nano-filtrated pdC1-INH (Cinryze^®^), has been used in fixed doses (500IU or 1,000IU), in lower severity attacks, such as angioedema of limbs and abdominal pain episodes.^(31)^ However, there is evidence that fixed doses may not be sufficient to control the attacks, and that the 20IU/kg dose is more effective. Bork et al.,^(31)^ observed that more than 60% of patients with a laryngeal edema who received fixed doses of C1-INH concentrate required another dose of the drug.

The recombinant human C1-INH (Ruconest^®^) is obtained from the purification of transgenic rabbit milk. Studies have demonstrated its efficacy and safety, with no adverse thrombotic events, though it should be used with care in those with allergy to rabbits.^(26)^ It is not available in Brazil, and the established dose is from 60IU to 100IU per kg of body weight, per dose.

Icatibant (Firazyr^®^) is a synthetic molecule, similar to bradykinin, and acts as a competitive and selective antagonist to the bradykinin B2 receptor. The safety and efficacy of icatibant have been demonstrated in three clinical trials - phases I, II, and III.^(32,33)^ Hereditary angioedema attacks are resolved more quickly with the early, rather than later use of icatibant, therefore, administration is recommended in the first 6 hours after onset of symptoms.^(34)^It is licensed in Brazil, including for administration at home. Home use is safe, and patients report only erythema and pain at the injection site, with spontaneous resolution.^(35)^ The recommended dose is 30mg for adults and 0.4mg/kg in the age range of 2 to 17 years, subcutaneously, exclusively in the abdominal region. Additional injections may be administered every 6 hours, up to a maximum of three injections in 24 hours.^(32)^

The fourth class of drugs to treat attacks is kallikrein inhibitors, such as Ecallantide (Kalbitor^®^), approved for use in the United States and not available in Brazil. The recommended dose is 30mg subcutaneously, but it is not approved for home administration, since anaphylaxis has been observed in approximately 3% of patients.^(36)^To date, there are no studies comparing the efficacy of these drugs in randomized clinical trials. We therefore suggest, for greater efficacy, using the available option, as soon as possible after the onset of the attack.

Another alternative, in use for many years, for C1-INH replacement is the infusion of fresh frozen plasma, two to four units for adults, and 10 mL per kg, for children. However, this strategy has not yet been tested for efficacy and safety in in HAE in clinical trials and should be reserved for situations in which no other drug for attacks is available.^(1)^In addition, plasma administration offers not only C1-INH replacement, but also the substrates on which this inhibitor acts, and may not have adequate efficacy and even aggravate the condition.

### Treatment of hereditary angioedema attack in special situations

#### Children and adolescents

To date, few strategies have been licensed for use in the pediatric population. In Brazil, Berinert^®^ is approved, without age restriction, at a dose of 20IU/kg, and icatibant has been recently approved for use as of 2 years of age.^(37,38)^

## Pregnancy, childbirth, postpartum and lactation

There are few studies on the safety and efficacy of drugs for treating attacks during pregnancy, puerperium, and lactation. The treatment of choice during pregnancy, childbirth, postpartum, and breastfeeding is the pdC1-INH concentrate.^(39)^ When pdC1-INH concentrate is not available, fresh frozen plasma can be administered.^(39)^

Icatibant (Firazyr^®^) and pdC1-INH concentrate (Berinert^®^) are classified as Category C by the Food and Drug Administration (FDA) for use in pregnancy and lactation. However, these drugs have been used during pregnancy in situations of neccessity, proving to be effective and safe, with no adverse effect for pregnant women and neonates.^(40)^

Patients seen in the emergency department with suspected HAE should be referred to a specialist for investigation. Cases with confirmed diagnosis should be guided as to the need for regular specialized follow-up to identify possible triggers of attacks. They should also be evaluated as to the need for long-term and short-term prophylactic treatment (invasive diagnostic procedures, surgeries, and dental treatments). Some drugs used for treatment of attacks can also be used in prophylaxis, such as pdC1-INH concentrate. Recently, an immunobiological agent has been approved that blocks kallikrein, and consequent bradykinin formation, indicated for long-term prophylaxis.^(41)^(Table 4).

**Table 4:** Drugs for short- and long-term prophylaxis used in the treatment of hereditary angioedema

Drug	Brand name	Mechanism of action	Indication	Dosage (prophylaxis)	Administration route	Side effects
Tranexamic acid	Generic, Hemoblock^®^ and Transamin^®^	Antifibrinolytic action	Long-term prophylaxis Short-term prophylaxis	20-50mg/kg/day (2-3 times/day) up to 4-6g/day; pills=250mg 25mg/kg/day (maximum dose 3-6g/day), 5 days before and 2-5 days after the procedure	Oral	Muscle pain, weakness, elevated CPK, nausea, diarrhea, vertigo, postural hypotension, severe fatigue and thrombosis
Danazol	Ladogal^®^	Attenuated androgen; increases levels of C1-INH	Long-term prophylaxis Short-term prophylaxis	Up to 200mg/day 10mg/kg/day (maximum dose of 600mg/day), for 5-7 days before 2 days after the procedure	Oral	Weight gain, headache, menstrual abnormalities, acne, libido modification, anxiety, mood disorders, hypertension, myopathy, changes in lipid profile, hematuria, hepatoma and hepatocarcinoma
Oxandrolone	Compounded	Attenuated androgen; increases the levels of C1-INH	Long-term prophylaxis	Up to 2.5mg, every 8-12 hours, up to 20mg/day Child: 0.25mg/kg/day	Oral	
pdC1-INH^*^ concentrate	Cinryze^®†^	C1-INH replacement	Long-term prophylaxis Short-term prophylaxis	1,000IU, every 3-4 days 500-1,000IU before the procedure	Intravenous	Thrombotic events were observed in some patients
pdC1-INH^*^ concentrate	Berinert^®‡^	C1-INH replacement	Long-term prophylaxis Short-term prophylaxis	20IU/kg, every 3-4 days 20IU/kg or 500-1,000IU before the procedure	Intravenous	
Lanadelumab^§^	Thakzyro^®^	Kallikrein inhibitor	Long-term prophylaxis	300mg every 4 weeks	Subcutaneous	Injection site reactions, hypersensitivity, dizziness, maculopapular eruption, myalgia and hepatic enzyme modifications

Source: adapted from Giavina-Bianchi P, Arruda LK, Aun MV, Campos RA, Chong-Neto HJ, Constantino-Silva RN, et al. Diretrizes brasileiras para o diagnóstico e tratamento do angioedema hereditário – 2017. Arq Asma Alerg Imunol. 2017;1(1):23-48.^(1)^ * Plasma-derived C1-INH concentrate; ^†^ Cinryze^®^ requires imports; ^‡^ Berinert^®^ was approved only for treatment of acute attacks in the United States and in Brazil; ^§^ Lanadelumab is a monoclonal antibody (IgG anti-kallikrein). CPK: creatine phosphokinase; C1-INH: C1 inhibitor.

## CONCLUSION

Hereditary angioedema attacks are unusual causes of angioedema in the emergency department. Restricted knowledge of hereditary angioedema by healthcare professionals can lead to incorrect diagnoses, especially when these attacks manifest in internal organs. Although rare, due to its potential for fatality, physicians acting in the emergency department should be aware of the warning signs of hereditary angioedema, especially when there is no response to treatment with usual drugs, such as antihistamines and corticosteroids. The correct and immediate management of these patients prevents unnecessary surgeries and, above all, fatal outcomes.
