# Supplementary material for: Hereditary angioedema: how to approach it at the emergency department?
Source: Einstein (Sao Paulo). 2021 Apr 1;19:eRW5498. doi: 10.31744/einstein_journal/2021RW5498 (PMC8020838; doi:10.31744/einstein_journal/2021RW5498)
Supplement: Translation

# Translation: Angioedema hereditário: como abordar na emergência?

**Authors:** Faradiba Sarquis Serpa, Eli Mansour, Marcelo Vivolo Aun, Pedro Giavina-Bianchi, Herberto José Chong, Luisa Karla Arruda, Regis Albuquerque Campos, Antônio Abílio Motta, Eliana Toledo, Anete Sevciovic Grumach, Solange Oliveira Rodrigues Valle

**Affiliations:** 1 Brasil Escola Superior de Ciências, Santa Casa de Misericórdia de Vitória, Vitória, ES, Brasil.; 2 Brasil Faculdade de Ciências Médicas, Universidade Estadual de Campinas, Campinas, SP, Brasil.; 3 Brasil Faculdade Israelita de Ciências da Saúde Albert Einstein, Hospital Israelita Albert Einstein, São Paulo, SP, Brasil.; 4 Brasil Faculdade de Medicina, Universidade de São Paulo, São Paulo, SP, Brasil.; 5 Brasil Universidade Federal do Paraná, Curitiba, PR, Brasil.; 6 Brasil Faculdade de Medicina de Ribeirão Preto, Universidade de São Paulo, Ribeirão Preto, SP, Brasil.; 7 Brasil Faculdade de Medicina, Universidade Federal da Bahia, Salvador, BA, Brasil.; 8 Brasil Faculdade de Medicina de São José do Rio Preto, São José do Rio Preto, SP, Brasil.; 9 Brasil Faculdade de Medicina do ABC, Santo André, SP, Brasil.; 10 Brasil Universidade Federal do Rio de Janeiro, Rio de Janeiro, RJ, Brasil.

**Keywords:** Angioedema, Angioedemas hereditários, Emergências, Inibidor de C1, Dor abdominal, Edema laríngeo, Asfixia, Bradicinina

## Abstract

As crises de angioedema são causas comuns de atendimentos nas emergências, e devido ao potencial de gravidade, é importante que os profissionais que atuam nesses serviços conheçam suas causas e abordagem. Os mecanismos envolvidos no angioedema sem urticas podem ser histaminérgicos ou mediados por bradicinina. As causas mais comuns de angioedema mediado por histamina são alimentos, medicamentos, ferroada de insetos e idiopática. Quando o mediador é a bradicinina, os desencadeantes são os inibidores da enzima conversora de angiotensina e fatores relacionados ao angioedema adquirido com deficiência do inibidor de C1 ou angioedema hereditário que são menos comuns, mas muito importantes pela possibilidade de desfecho fatal. O angioedema hereditário é uma doença rara, caracterizada por crises de edema que acometem o tecido subcutâneo e mucosas de vários órgãos, manifestando-se principalmente por crises de angioedema e dor abdominal. Esse tipo de angioedema não responde ao tratamento usual com adrenalina, anti-histamínicos e corticosteroides. Assim, se não identificados e tratados adequadamente, esses pacientes têm risco de morte por edema de laringe estimado em 25% a 40%. O tratamento do angioedema hereditário mudou drasticamente nos últimos anos, com o desenvolvimento de novos e eficientes fármacos para as crises: inibidor de C1 derivado de plasma, inibidor de C1 recombinante humano, antagonista do receptor B2 da bradicinina (icatibanto) e o inibidor da calicreína (ecalantide). No Brasil, até o momento, estão liberados para uso o inibidor de C1 derivado de plasma e o icatibanto. O manejo correto desses pacientes na emergência evita cirurgias desnecessárias e, principalmente, desfechos fatais.

## INTRODUÇÃO

O angioedema caracteriza-se por edema localizado, não inflamatório, assimétrico, desfigurante e autolimitado da derme profunda, tecidos subcutâneo e/ou submucoso, decorrente da vasodilatação e do aumento da permeabilidade vascular.^(1)^

Devido a potencial gravidade, principalmente quando o angioedema acomete as vias aéreas, a abordagem terapêutica na emergência é, muitas vezes, realizada logo após a admissão, antes da anamnese e da possível identificação da etiologia. Entretanto, o reconhecimento e a diferenciação das diversas etiologias e patogênese do angioedema são importantes para um tratamento eficaz na emergência.^(2,3)^

O mecanismo envolvido no angioedema sem urticas pode ser mediado por histamina ou bradicinina. As causas mais comuns de angioedema histaminérgico são alimentos, medicamentos, ferroada de insetos e idiopática. No angioedema mediado por bradicinina, os desencadeantes mais frequentes são os inibidores da enzima conversora de angiotensina (iECA) e fatores relacionados ao angioedema hereditário (AEH) ou angioedema adquirido por deficiência do inibidor de C1 (AEA). Embora menos comuns, o AEA e o AEH são relevantes, pela possibilidade de desfecho fatal, caso uma terapêutica inapropriada seja adotada.^(4)^

O AEH é uma doença rara, potencialmente fatal e ainda subdiagnosticada, cujas crises podem acometer tanto a derme e o subcutâneo, quanto órgãos internos – predominantemente o trato gastrintestinal e as vias aéreas superiores –, sendo o desfecho mais temido o angioedema laríngeo. Estima-se que 25% a 40% das crises de AEH evoluem para óbito por asfixia, quando não tratadas.^(5)^ O edema de alças intestinais é frequente e incapacitante, e a dor abdominal pode ser a única manifestação durante a crise. Pode ocorrer o diagnóstico equivocado de abdome agudo ocasionando cirurgia desnecessária.^(6)^

Na emergência, as crises de AEH podem ser confundidas com outros tipos de angioedema, principalmente o histaminérgico. Estudo brasileiro mostrou que 29% dos pacientes com AEH relataram óbitos em familiares por asfixia.^(7)^ Na casuística de Bork et al.,^(5)^ em um terço dos pacientes que foram a óbito, a causa foi asfixia, sendo o risco maior naqueles sem diagnóstico confirmado.

Dados dos Estados Unidos mostram que, anualmente, ocorrem aproximadamente 110 mil visitas às emergências por AEH ou AEA, e esses pacientes apresentam taxa de admissão hospitalar maior em relação aos atendidos por angioedema de causa alérgica.^(8)^Em estudo italiano, 0,37% dos pacientes admitidos nas emergências durante 6 meses foram diagnosticados com angioedema por causas diversas.^(9)^No Canadá, 0,1% dos atendimentos nas emergências foram por angioedema de diferentes etiologias, e mais de um terço dos pacientes relataram visitas prévias pela mesma causa.^(10)^ No Brasil, não há dados disponíveis sobre o número de atendimentos por crises de angioedema nas emergências.

Considerando-se a necessidade de melhorar o conhecimento sobre o AEH por parte dos profissionais de saúde, especialmente os que atuam em emergências, os autores abordam os aspectos relevantes para o atendimento das crises de angioedema.

## MÉTODOS

Foram avaliadas publicações que abordassem o tema AEH por meio de revisão em bases de dados MEDLINE^®^ (PubMed^®^), Cochrane e *Scientific Electronic Library Online* (SciELO), e considerados os consensos e diretrizes previamente publicados, com a finalidade de utilizar práticas já aprovadas por *experts* no assunto, inclusive em nosso país, nos últimos 10 anos. Foram utilizados os Descritores em Ciências da Saúde (DeCS) “*hereditary angioedema*”, “*emergency*” e “*bradykinin*”, o operador booleano *AND* e suas correspondentes em português.

### Classificação do angioedema sem urticas

O angioedema pode ser classificado em relação ao mediador vasoativo causador do edema, histamina ou bradicinina. Essa classificação permite identificar o tipo de angioedema e orienta quanto ao tratamento adequado^(3)^ (Figura 1).

**Figura 1:** Classificação do angioedema quanto aos mecanismos e defeitos associados

C1-INH: inibidor de C1; FXII: fator XII da coagulação; ANGPT1: angiopoetina-1; PLG: plasminogênio; KNG1: cininogênio 1; ECA: enzima conversora da angiotensina; ATII: angiotensina II; IgE: imunoglobulina E; AINEs: anti-inflamatórios não esteroidais.

O angioedema histaminérgico é o mais frequente. Tem evolução rápida,^(4)^ é geralmente associado com urticas e ocorre por desgranulação de mastócitos, mediada ou não por imunoglobulina E (IgE), ou por alteração no metabolismo do ácido araquidônico, como nos casos induzidos por anti-inflamatórios não esteroidais (AINEs).^(11)^

Os casos mediados por bradicinina caracterizam-se clinicamente pela ausência de urticas e incluem o AEH, o AEA-inibidor de C1 (C1-INH) e o induzido por iECA, bloqueador do receptor de angiotensina II e gliptinas.

O AEH tem dois subtipos: por deficiência do inibidor de C1 (AEH C1-INH) e com C1-INH normal (AEH com C1-INH normal).^(1,12)^ A herança genética do AEH C1-INH é autossômica dominante, decorrente de mutações no gene SERPING1, que codifica uma serinoprotease, o C1-INH, com vários locais de atuação. Tais mutações resultam em deficiência quantitativa e/ou funcional de C1-INH, o que ocasiona a produção exagerada de bradicinina, com consequente aumento da permeabilidade vascular e surgimento de angioedema. O AEH com C1-INH normal está associado a outras mutações, e também resulta da produção exagerada de bradicinina.^(13,14)^ O maior número de casos descritos é de mutação no fator XII da coagulação (FXII).^(13,14)^ Outras mutações recentemente descritas são nos genes do plasminogênio (AEH-PLG),^(15)^ da angiopoietina-1 (AEH-ANGPT1),^(13)^ e do cininogênio 1 (KNG1).^(16)^

O AEA-C1-INH ocorre devido ao consumo de C1-INH, e as causas mais comuns são as doenças linfoproliferativas e autoimunes.^(17)^O quadro clínico é semelhante ao AEH, mas o início dos sintomas é tardio, e a história familiar é negativa.^(1,12)^

Os anti-hipertensivos do grupo de iECA são causa frequente de atendimentos por angioedema nas emergências e representam 0,1% a 0,5% desses casos.^(17)^ As crises podem ocorrer independentemente do tempo de uso, e os iECA mais relacionados à ocorrência de angioedema são o captopril, o enalapril e o lisinopril.^(18)^ Os medicamentos do grupo das gliptinas, sitagliptina, vildagliptina, saxagliptina, alogliptina e linagliptina, utilizados no tratamento do *diabetes mellitus* tipo 2, também são potenciais desencadeantes de angioedema. Esses fármacos inibem a enzima dipeptidil peptidase 4 (DPP-4), que é importante na degradação da bradicinina e da substância P.^(19)^

Dados sobre a prevalência de angioedema mediado por bradicinina são escassos, e estudos mostram que o angioedema por iECA é mais comum que o AEH, e, por sua vez, este é mais comum que o AEA-C1-INH.^(20)^

Algumas características clínicas, como, por exemplo, idade de início dos sintomas, história familiar positiva, urticas associadas, localização da crise e velocidade de instalação, permitem diferenciar os cinco principais fenótipos de angioedema (Tabela 1).

**Tabela 1:** Características clínicas dos principais fenótipos de angioedema

Características clínicas	Principais fenótipos de angioedema					
	Angioedema histaminérgico ou alérgico	AEH com deficiência de C1-INH	AEH com C1-INH normal	AEA-C1-INH	iECA	Idiopático
Idade de início	Variável	Infância	Adolescente/adulto	Adulto	Adulto	Variável
História familiar	Atopia (?)	75% casos	Variável	Não	Não	Não
Predileção por sexo	Não	Não	Feminino	Não	Não	Não
Urticas associadas	Sim/não	Não	Não	Não	Não	Sim/não
Local da crise	Lábios, olhos, língua e laringe	Face, língua, laringe, genital, extremidades e abdome	Face, língua, laringe, genital, extremidades e abdome	Face, língua, laringe, genital, extremidades e abdome	Face, língua e laringe	Lábios, olhos, língua e laringe
Velocidade de instalação da crise	Rápida (até 1 hora)	Lenta e gradual (horas)	Lenta e gradual (horas)	Lenta e gradual (horas)	Lenta e gradual (horas)	Variável

Fonte: adaptado de Moellman JJ, Bernstein JA, Lindsell C, Banerji A, Busse PJ, Camargo CA Jr, Collins SP, Craig TJ, Lumry WR, Nowak R, Pines JM, Raja AS, Riedl M, Ward MJ, Zuraw BL, Diercks D, Hiestand B, Campbell RL, Schneider S, Sinert R; American College of Allergy, Asthma & Immunology (ACAAI); Society for Academic Emergency Medicine (SAEM). A consensus parameter for the evaluation and management of angioedema in the emergency department. Acad Emerg Med. 2014;21(4):469-84.^(21)^ AEH: angioedema hereditário; C1-INH: inibidor de C1; AEA-C1-INH: angioedema adquirido com deficiência do inibidor de C1; iECA: inibidor da enzima conversora de angiotensina.

### Fisiopatologia do angioedema mediado por bradicinina

A bradicinina é o principal mediador no AEA e no AEH com deficiência do C1-INH quantitativa e/ou funcional. O C1-INH regula várias proteínas dos sistemas do complemento, de contato, coagulação e fibrinolítico. A ativação do sistema de contato inicia-se com o FXII ativado, que converte a pré-calicreína em calicreína; esta cliva o cininogênio de alto peso molecular (HK), levando à liberação da bradicinina. Essa cinina liga-se ao seu receptor B2, que é constitutivamente expresso em células endoteliais e interfere nas junções endoteliais, aumentando a permeabilidade vascular e provocando angioedema. Na deficiência do C1-INH, a falta de controle da ativação do sistema de contato é mais relevante para a liberação da bradicinina. Por outro lado, por ativação do sistema complemento, o nível plasmático de C4 diminui por falta do C1-INH, sendo a dosagem de C4 utilizada como exame de triagem para o AEH^(1,22)^ (Figura 2).

**Figura 2:** Fisiopatologia do angioedema sem urticas mediado por histamina (A) e por bradicinina (B)

Fonte: adaptado de Bernstein JA, Cremonesi P, Hoffmann TK, Hollingsworth J. Angioedema in the emergency department: a practical guide to differential diagnosis and management. Int J Emerg Med. 2017;10(1):15. Review.^(3)^

Em relação ao AEH com C1-INH normal, as mutações no FXII resultam em ativação maior do FXII pela plasmina, gerando o fator XII ativado, que atua na pré-calicreína.^(13,14)^ Dessa forma, o FXII mutante ativa-se rapidamente após clivagem pela plasmina e escapa da inibição de C1-INH. Nos casos de AEH com mutações nos genes PLG,^(15)^ ANGPT1^(13)^ou KNG1,^(16)^ outros mecanismos estão envolvidos. O PLG, por exemplo, é um zimogênio circulante, que é convertido na enzima ativa plasmina por clivagem de uma ligação peptídica. A plasmina é a principal enzima da fibrinólise, que parece estar envolvida na patogênese do AEH com C1-INH normal, mas o papel do sistema fibrinolítico no AEH ainda não está totalmente esclarecido.^(15)^ No caso das mutações no gene da ANGPT1, é descrita a perda da função da proteína, com consequente redução da capacidade da ativação do receptor tirosina-quinase 2 (TIE2), expresso em células endotelais. Este, por sua vez, atuaria na menor permeabilidade vascular.^(13)^ Na mutação no gene do KNG1, ocorre formação de uma bradicinina aberrante, com meia-vida mais prolongada e, consequentemente, mais ativa.^(16)^

### Diagnóstico clínico-laboratorial

Na abordagem do paciente com angioedema, a história clínica detalhada, os aspectos do exame físico e a resposta ao tratamento convencional com anti-histamínicos (anti-H1), corticosteroides (CE) e adrenalina podem indicar a possível etiologia.

A presença de urticas associadas ao angioedema, tanto no episódio atual como em crises prévias, sugere fortemente angioedema histaminérgico.^(17)^ Esse tipo de angioedema evolui rapidamente,^(6)^ e os desencadeantes mais comuns são medicamentos, alimentos, veneno de insetos, fatores físicos (calor, frio e pressão) ou, ainda, idiopáticos. Nessas situações, a remoção do fator desencadeante e o tratamento convencional são eficazes.^(17)^

Quando houver acometimento de outros órgãos ou sistemas, configurando anafilaxia, a dosagem da triptase sérica pode corroborar essa hipótese. Esse exame geralmente não está disponível nos serviços de emergência brasileiros, e a demora para o resultado não permite auxiliar a abordagem na emergência, porém, um nível elevado de triptase durante a crise confirma desgranulação dos mastócitos. Por outro lado, a dosagem normal de triptase na crise não exclui anafilaxia.^(21)^ A triptase elevada não ocorre no angioedema induzido por bradicinina, como AEH, AEA-C1-INH ou induzido por iECA.

As crises de angioedema induzidas por iECA, AEA-C1-INH e AEH, todas mediadas por bradicinina, costumam levar horas até a instalação completa, mas existe descrição de quadros súbitos.^(21)^ Além disso, não apresentam urticas associadas e não respondem ao tratamento convencional.^(1,17)^

Quanto ao AEH, o diagnóstico geralmente não é feito no atendimento da emergência. Alguns dados clínicos contribuem para o diagnóstico, como antecedente familiar de angioedema, edemas que persistem por 3 a 5 dias, não resposta ao tratamento convencional, edema de via aérea recorrente, crises de dor abdominal sem causa aparente, por vezes, com história de cirurgias abdominais não terapêuticas (“laparotomias brancas”).^(21)^ Em alguns casos, podem ocorrer sinais e sintomas prodrômicos, como o eritema marginado, caracterizado por lesões eritematosas, serpinginosas e não pruriginosas, que podem ser confundidas com urticas.^(1)^ A história familiar de AEH é um sinal de alerta importante, mas está ausente em aproximadamente 25% dos casos.^(17)^

Outro dado que chama a atenção nos portadores de AEH é que as mulheres costumam ter maior frequência e/ou gravidade de crises em fases de maior nível estrogênico, seja endógeno (ciclo menstrual, gestação), seja sob administração exógena (contraceptivos ou reposição hormonal).^(14)^ O AEH com níveis normais de C1-INH está mais associado ao estrógeno, sendo ainda mais frequente em mulheres.^(14)^

Pacientes sem outra causa de angioedema, com algum desses achados, devem ser abordados como possíveis portadores de AEH, principalmente se não houver resposta clínica ao tratamento convencional com adrenalina, anti-H1 e CE.^(17)^

O diagnóstico do AEH C1-INH é confirmado por avaliação quantitativa e/ou funcional do C1-INH. Exames laboratoriais não são indispensáveis para o diagnóstico na emergência. Entretanto, a dosagem de C4 durante a crise pode contribuir para o diagnóstico. Estima-se que quase 100% desses pacientes tenham redução dos níveis de C4 durante a crise, ou seja, o C4 normal na crise permite excluir AEH C1-INH.^(21)^

O AEA-C1-INH pode ser diferenciado do AEH C1-INH por meio da dosagem de C1q, que se encontra em níveis baixos, além da redução de C4, C1-INH quantitativo e funcional.

### Sinais de alerta para angioedema hereditário

Grumach et al.,^(23)^ elegeram os principais sinais de alerta para o AEH, baseados na fisiopatologia, sinais e sintomas, exames laboratoriais e resposta a terapias específicas. Para facilitar a memorização, os sinais foram listados em ordem alfabética e chamados de ABC do angioedema.^(23)^

Ainda, com o mesmo objetivo, o Grupo de Estudos Brasileiro em Angioedema Hereditário (Gebraeh) criou uma regra mnemônica com os sinais de alerta para o diagnóstico do AEH: HAAAAE.^(1)^ Todos esses sinais estão listados na tabela 2.^(1)^

**Tabela 2:** Sinais de alerta para o angioedema hereditário

HAAAAE^(1)^	ABC do angioedema^(23)^
Hereditariedade	Angioedema
Angioedema recorrente	Bradicinina
Dor abdominal	C1 inibidor
Ausência de urticas	Desencadeantes
Anti-histamínicos sem efeito	Epinefrina sem resposta
Associação com estrógeno	Familiares afetados
	Gastrintestinal e/ou edema de glote

Fonte: Giavina-Bianchi P, Arruda LK, Aun MV, Campos RA, Chong-Neto HJ, Constantino-Silva RN, et al. Diretrizes brasileiras para o diagnóstico e tratamento do angioedema hereditário – 2017. Arq Asma Alerg Imunol. 2017;1(1):23-48;^(1)^ Grumach AS, Ferraroni N, Olivares MM, López-Serrano MC, Bygum A. An ABC of the warning signs of hereditary angioedema. Int Arch Allergy Immunol. 2017;174(1):1-6. Review.^(23)^

### Desencadeantes das crises de angioedema hereditário

As crises de AEH podem ser espontâneas ou desencadeadas por fatores identificados por meio de história clínica detalhada. São descritos como desencadeantes: trauma, estresse, infecção, procedimento cirúrgico e dentário, menstruação, gestação, consumo de bebida alcoólica, mudança extrema de temperatura, exercício físico, uso de iECA, exposição ao estrógeno, entre outros.^(1)^ A identificação do fator desencadeante pode contribuir para o diagnóstico e seguimento futuro.

### Tratamento da crise de angiodema hereditário

O primeiro passo da abordagem ao paciente em crise de AEH acometendo as vias aéreas, lingua e/ou úvula é manter a via aérea pérvia. Em pacientes instáveis, com risco iminente de asfixia, não se deve postergar a intubação orotraqueal (IOT)^(21)^ (Figura 3). É importante ressaltar que, na fase inicial de obstrução das vias aéreas, não se observa queda da saturação de oxigênio. A monitorização em sala de emergência está indicada e, em casos de hipotensão ou desidratação, a reposição de fluidos deve ser aplicada.^(24)^

**Figura 3:** Algoritmo para abordagem do angioedema agudo no serviço de emergência

IgE: imunoglobulina E; Fator Xlla: fator XII ativado; Recptor B2: receptor de bradicinina.

Anti-H1: anti-histamínico; CE: corticosteroide; AEH: angioedema hereditário; AEA: angioedema adquirido; iECA: inibidor da enzima conversora de angiotensina.

O tratamento da crise no paciente com diagnóstico de AEH pode variar com a gravidade e a localização da crise e envolve o uso de medicamentos que atuam impedindo a ação da bradicinina nas células endoteliais ou aumentando os níveis do C1-INH, e, consequentemente, reduzindo os níveis de bradicinina^(1)^(Figura 4).

**Figura 4:** Recomendações para tratamento da crise de angioedema hereditário, de acordo com a área afetada

* Disponíveis no Brasil: concentrado de inibidor de C1 (Berinert^®^) e icatibanto (Firazyr^®^); ^#^ em todas as situações, caso não esteja disponível o concentrado do inibidor de C1 ou o icatibanto, usar o plasma fresco congelado.

O tratamento medicamentoso mudou drasticamente nos últimos anos, com novos e eficientes fármacos para o manejo das crises. Há quatro grupos de drogas disponíveis: concentrado do inibidor de C1 derivado de plasma (pd C1-INH), inibidor de C1 recombinante humano, (Rh C1-INH), antagonista do receptor B2 da bradicinina (icatibanto) e inibidor da calicreína (ecalantide).^(24-26)^ No Brasil, até o momento, há dois produtos aprovados pela Agência Nacional de Vigilância Sanitária (ANVISA): o pdC1-INH (Berinert^®^) e o icatibanto (Firazyr^®^)^(1,27,28)^ (Tabela 3).

**Tabela 3:** Características e orientações para os medicamentos disponíveis para crise no Brasil

Características/orientações	Medicamento	
	Icatibanto (Firazyr^®^)	Concentrado C1-INH (Berinert^®^)
Faixa etária	>2 anos	Sem limite de idade
Apresentação	10mg/mL de icatibanto (seringa com 3mL da solução)	500UI de C1-INH em pó liofilizado
Dose	0,4mg/kg até 18 anos 30mg acima de 18 anos	20UI/kg
Via de administração	Subcutânea, lentamente Preferencialmente na região abdominal	Intravenosa lenta ou infusão (4mL/minuto)
Aspecto da solução	Incolor e clara	Incolor e límpida
Temperatura de armazenamento	2°C a 8°C	2°C a 8°C
Armazenamento após uso de dose fracionada ou reconstituição	Não recomendado Abaixo 18 anos e de 65kg, o volume não utilizado deverá ser desprezado	Após reconstituição, exclusivamente no frasco ampola
Tempo de armazenamento	Não recomendado	Máximo 8 horas em temperatura ambiente

Fonte: Brasil. Ministério da Saúde. Agência Nacional de Vigilância Sanitária (ANVISA). Bulário eletrônico. Firazyr: solução injetável. Bula profissional do medicamento. Brasília (DF): ANVISA; 2019 [citado 2020 Jan 22]. Disponível em: https://consultas.anvisa.gov.br/#/bulario/q/?nomeProduto=FIRAZYR;^(27)^ Brasil. Ministério da Saúde. Agência Nacional de Vigilância Sanitária (ANVISA). Bulário eletrônico. Berinert: pó liofilizado para solução injetável. Bula profissional do medicamento. Brasília (DF): ANVISA;2019 [citado 2020 Jan 22]. Disponível em: https://consultas.anvisa.gov.br/#/bulario/q/?nomeProduto=berinert^(28)^ C1-INH: inibidor de C1.

Os concentrados de C1-INH para uso intravenoso demonstraram ser eficazes e seguros no tratamento de todas as formas de crises de AEH por deficiência do C1-INH em crianças e adultos.^(25,29,30)^ O Berinert^®^, aprovado no Brasil, é indicado para administração por via intravenosa na dose de 20UI/kg, independente da gravidade da crise.^(29)^ Outro concentrado de pdC1-INH nanofiltrado (Cinryze^®^), foi utilizado em doses fixas (500UI ou 1.000UI), em crises de menor gravidade, como no angioedema de extremidades e nas crises abdominais.^(31)^ Entretanto, existem evidências de que doses fixas podem não ser suficientes para controlar as crises, e a dose de 20UI/kg é mais eficaz. Bork et al.,^(31)^ observaram que mais de 60% dos pacientes com crise de edema de laringe que receberam doses fixas de concentrado de C1-INH necessitaram de nova dose do medicamento.

O C1-INH recombinante humano (Ruconest^®^) é obtido a partir da purificação de leite de coelhas transgênicas. Estudos demonstraram sua eficácia e segurança, sem eventos adversos trombóticos, com recomendação de cuidado para seu uso em alérgicos a coelhos.^(26)^ Não está disponível no Brasil, e a dose estabelecida é de 60UI a 100UI por kg de peso corporal, por dose.

O icatibanto (Firazyr^®^) é uma molécula sintética, semelhante à bradicinina, e age como antagonista competitivo e seletivo do receptor B2 da bradicinina. A segurança e a eficácia do icatibanto foram demonstradas em três ensaios clínicos de fases I, II e III.^(32,33)^ As crises de AEH são resolvidas mais rapidamente com o uso precoce do icatibanto em comparação com o uso tardio. Por isso, recomenda-se a administração nas primeiras 6 horas após o início dos sintomas.^(34)^ No Brasil, está licenciado, inclusive para autoadministração domiciliar. O uso domiciliar é seguro, e os pacientes relatam apenas eritema e dor no local da injeção, com resolução espontânea.^(35)^ A dose preconizada é de 30 mg para adultos e de 0,4mg/kg na faixa etária de 2 a 17 anos, por via subcutânea, exclusivamente na região abdominal, podendo ser administradas injeções adicionais, a cada 6 horas, até o máximo de três injeções em 24 horas.^(32)^

A quarta classe de drogas para tratamento das crises é a dos inibidores da calicreína, cujo representante é o Ecallantide (Kalbitor^®^), aprovado para utilização nos Estados Unidos e indisponível no Brasil. A dose recomendada é de 30mg por via subcutânea, e não está aprovado para autoadministração domiciliar, pois a anafilaxia foi observada em aproximadamente 3% dos pacientes.^(36)^ Até o momento, não existem estudos comparando a eficácia desses fármacos em ensaios clínicos randômicos. Portanto, sugerimos usar a opção disponível, e com menor tempo possível entre o início da crise e sua aplicação, para melhor eficácia.

Outra alternativa de reposição de C1-INH que é usada há muitos é a infusão de plasma fresco congelado, na dose de duas a quatro unidades, para adultos, e de 10mL/kg, para crianças. Contudo, essa estratégia não foi testada em ensaios clínicos quanto à eficácia e à segurança no AEH e, portanto, deve ficar reservada para situações nas quais nenhuma outra droga para crises esteja disponível.^(1)^ Além disso, a administração do plasma oferece não somente a reposição do C1-INH, mas também os substratos nos quais essa protease atua, podendo não ter eficácia adequada e, inclusive, agravar o quadro.

### Tratamento da crise de angioedema hereditário em situações especiais

#### Crianças e adolescentes

Até o momento, poucas estratégias foram licenciadas para uso em população pediátrica. No Brasil, o Berinert^®^ está aprovado, sem restrição de faixa etária, na dose de 20UI/kg, e o icatibanto foi aprovado recentemente para uso a partir dos 2 anos de idade.^(37,38)^

## Gravidez, parto, pós-parto e lactação

Há poucos estudos sobre segurança e eficácia das drogas para tratamento das crises durante gestação, puerpério e lactação. O tratamento de escolha durante gravidez, parto, pós-parto e amamentação é o concentrado de pdC1-INH.^(39)^ Quando o concentrado de pdC1-INH não estiver disponível, o plasma fresco congelado pode ser administrado.^(39)^

O concentrado do C1-INH (Berinert^®^) e o icatibanto (Firazyr^®^) são classificados como categoria C do *Food and Drug Administration* (FDA) para uso na gravidez e na lactação. Entretanto, esses medicamentos têm sido usados durante a gestação em situações nas quais se fazem necessários, mostrando-se eficazes e seguros, sem qualquer consequência para as gestantes e os neonatos.^(40)^

Os pacientes atendidos na emergência com suspeita de AEH devem ser encaminhados ao especialista para investigação. Os casos com diagnóstico confirmado devem ser orientados quanto à necessidade de acompanhamento especializado regular, para identificação de possíveis desencadeantes de crises. Os mesmos também devem ser avaliados quanto à necessidade de tratamento profilático de longo e curto prazo (procedimentos diagnósticos invasivos, cirurgias e tratamentos dentários). Alguns medicamentos utilizados para o tratamento das crises também podem ser usados na profilaxia, como, por exemplo, o concentrado do pdC1-INH. Recentemente, foi aprovado um imunobiológico que bloqueia a calicreína e, consequentemente, a formação de bradicinina, indicado para profilaxia de longo prazo^(41)^(Tabela 4).

**Tabela 4:** Medicamentos para profilaxia de curto e longo prazo utilizados no tratamento do angioedema hereditário

Medicamento	Nome de marca	Mecanismo de ação	Indicação	Dosagem (profilaxia)	Via de administração	Efeitos colaterais
Ácido tranexâmico	Genérico, Hemoblock^®^ e Transamin^®^	Ação antifibrinolítica	Profilaxia de longo prazo Profilaxia de curto prazo	20-50mg/kg/dia (em 2-3 vezes/dia) até 4-6g/dia; pílulas=250mg 25mg/kg/dia (dose máxima 3-6g/dia), 5 dias antes e 2-5 dias após o procedimento	Oral	Dor muscular, fraqueza, CPK elevada, náuseas, diarreia, vertigem, hipotensão postural, fadiga grave e trombose
Danazol	Ladogal^®^	Andrógeno atenuado; aumenta os níveis de C1-INH	Profilaxia de longo prazo Profilaxia de curto prazo	Até 200mg/dia 10mg/kg/dia (dose máxima de 600mg/dia), por 5-7 dias antes e 2 após o procedimento	Oral	Ganho de peso, cefaleia, alterações menstruais, acne, alterações na libido, ansiedade, transtornos de humor, hipertensão, miopatia, alterações no perfil lipídico, hematúria, hepatoma e hepatocarcinoma
Oxandrolona	Formulado	Andrógeno atenuado; aumenta os níveis de C1-INH	Profilaxia de longo prazo	Até 2,5mg, a cada 8-12 horas, até 20mg/dia Criança: 0,25mg/kg/dia	Oral	
Concentrado de pdC1-INH^*^	Cinryze^®†^	Reposição de C1-INH	Profilaxia de longo prazo Profilaxia de curto prazo	1.000UI, a cada 3-4 dias 500-1.000UI antes do procedimento	Intravenoso	Eventos trombóticos foram observados em alguns pacientes
Concentrado de pdC1-INH^*^	Berinert^®‡^	Reposição de C1-INH	Profilaxia de longo prazo Profilaxia de curto prazo	20UI/kg, a cada 3-4 dias 20UI/kg ou 500-1.000UI antes do procedimento	Intravenoso	
Lanadelumab^§^	Thakzyro^®^	Inibidor da calicreína	Profilaxia de longo prazo	300mg a cada 4 semanas	Subcutânea	Reações no local de injeção, hipersensibilidade, tontura, errupção maculopapular, mialgia e alteração de enzimas hepáticas

Fonte: adaptado de Giavina-Bianchi P, Arruda LK, Aun MV, Campos RA, Chong-Neto HJ, Constantino-Silva RN, et al. Diretrizes brasileiras para o diagnóstico e tratamento do angioedema hereditário – 2017. Arq Asma Alerg Imunol. 2017;1(1):23-48.^(1)*^ Concentrado de C1-INH derivado de plasma; ^†^ o medicamento Cinryze^®^ precisa ser importado; ^‡^ o medicamento Berinert^®^ foi aprovado apenas para o tratamento de crises agudas nos Estados Unidos e no Brasil; ^§^ o medicamento lanadelumab é um anticorpo monoclonal (IgG anticalicreína). CPK: creatinofosfoquinase; C1-INH: inibidor de C1.

## CONCLUSÃO

As crises de angioedema hereditário são causas pouco comuns de angioedema na emergência. O conhecimento restrito sobre o angioedema hereditário por parte dos profissionais de saúde pode levar ao diagnóstico incorreto, principalmente quando essas crises se manifestam em órgãos internos. Apesar de raro, devido ao seu potencial de gravidade, os médicos que atuam na emergência devem estar atentos aos sinais de alerta do angioedema hereditário, principalmente quando não há resposta ao tratamento com drogas usuais, como anti-histamínicos e corticosteroides. O manejo correto e imediato desses pacientes evita cirurgias desnecessárias e, principalmente, desfechos fatais.
